# Computational Environmental Impact Assessment of an Enhanced PVC Production Process

**DOI:** 10.3390/polym17243316

**Published:** 2025-12-16

**Authors:** Arelmys Bustamante Miranda, Segundo Rojas-Flores, Ángel Darío González-Delgado

**Affiliations:** 1 Research Group in Nanomaterials and Computer-Assisted Engineering (NIPAC), Chemical Engineering Program, Faculty of Engineering, University of Cartagena, Avenida del Consulado #Calle 30 No. 48 152, Cartagena de Indias 130015, Colombia; abustamantem@unicartagena.edu.co; 2 Institutos y Centros de Investigación, Universidad Cesar Vallejo, Trujillo 13001, Peru; srojasf@ucv.edu.pe

**Keywords:** suspension polymerization, environmental analysis, zero discharge, computer-aided process engineering

## Abstract

Poly(vinyl chloride) (PVC) is one of the most widely used polymers due to its strength, low cost, and light weight. Industrial production is mainly conducted by suspension polymerization, which facilitates the control of the emissions of vinyl chloride monomer (VCM), a known carcinogen. However, the process consumes large amounts of water and energy and generates residual compounds such as polyvinyl alcohol (PVA) and polymerization initiators, which must be properly managed to mitigate environmental impacts. To improve sustainability, this study applied mass- and energy-integration strategies together with a zero-liquid-discharge (ZLD) water-regeneration system that uses sequential aerobic and anaerobic reactors to recirculate process water with reduced PVA. Although these measures reduce resource consumption, they can displace or intensify other impacts; therefore, a comprehensive evaluation of the system is necessary. Accordingly, the objective of this study is to quantify and compare the potential environmental impacts (PEIs) of the improved PVC production process through a scenario-based assessment using a waste reduction algorithm (WAR). This is applied to four operating scenarios in order to identify the stages and flows that contribute most to the environmental burden. According to our literature review, there is limited published evidence that simultaneously combines mass/energy integration and a ZLD system in PVC processes; thus, this work provides an integrated assessment useful for industrial design. The environmental performance of the improved process was evaluated using WAR GUI software (v 1.0.17, which quantifies PEIs in categories such as toxicity, climate change, and acidification. Four scenarios were compared: Case 1 (excluding both product and energy), Case 2 (product only), Case 3 (energy only), and Case 4 (product and energy). The total PEI increased from 2.46 PEI/day in Case 1 to 6230 PEI/day in Case 4, with the largest contributions from acidification (5140 PEI/day) and global warming (496 PEI/day), mainly due to natural gas consumption (5184 GJ/day). In contrast, Cases 1 and 2 showed negative PEI values (−3160 and −2660 PEI/day), indicating that converting the toxic VCM (LD_50_: 500 mg/kg; ATP: 26 mg/L) into PVC (LD_50_: 2000 mg/kg; ATP: 100 mg/L) can reduce the environmental burden in certain respects. In addition, the ZLD system contributed to maintaining low aquatic toxicity in Case 4 (90.70 PEI/day).

## 1. Introduction

Poly(vinyl chloride) (PVC) is one of the most widely used thermoplastics in the world, alongside propylene and polyethylene. This popularity stems from its low density, outstanding chemical and mechanical resistance, biological inertness, fire and weather resistance, excellent durability, and good transparency. In addition, PVC is non-toxic in its stable form, making it ideal for medical applications, and serves as an effective barrier against gasses, moisture, and odors. Its impermeability and self-extinguishing properties further render it the material of choice across diverse sectors such as construction, automotive, food packaging, medicine, electronics and electrical, agriculture, and more [1].

Although PVC’s origins date back to a 1913 patent and it gained prominence after 1930, it has since become the second most-produced thermoplastic resin in the world, trailing only the polyolefins [2]. Throughout its development, PVC has driven advances in suspension and emulsion polymerization, stabilization chemistry, material modification, and processing techniques. Yet its versatility and popularity have also attracted intense criticism, particularly due to its reliance on chlorine chemistry and its high energy and water consumption. While many critiques emphasize the chlorine linkage as inherently unsustainable, some of these arguments rely more on emotional appeal than on rigorous technical or scientific analysis [3].

Despite its many advantages and the fact that, today, it is an almost indispensable material, the PVC life cycle generates environmental and health risks that must not be overlooked. On one hand, the production of chlorine, an essential raw material for the synthesis of vinyl chloride monomer (VCM), consumes approximately 1% of the world’s electricity, indicating a large energy footprint, and when produced by mercury cells, it releases this neurotoxic metal into the environment [4]. On the other hand, during suspension polymerization, which accounts for more than 80% of PVC production, the process requires an aqueous phase using polyvinyl alcohol (PVA) as a dispersant and consumes roughly ${3\;m}^{3}$ of water per tonne of PVC produced, while large-scale industrial plants may discharge just over ${100\;m}^{3}/h$ f effluent, directly impacting water resources [5]. In addition, from the synthesis of ethylene dichloride (EDC) and VCM to the accidental or controlled incineration of PVC, dangerous substances such as dioxins, furans, and other persistent, toxic, and bioaccumulative organochlorines, along with phthalates and metallic stabilizers (Pb and Cd), are formed, increasing environmental and health risks and impacts [6]. Together, these toxic byproducts affect not only the atmosphere but also soils, water bodies, and food chains, reinforcing the urgent need to evaluate and reduce the environmental impact of PVC production. Apart from the impacts at the manufacturing stage, the wide range of PVC applications also raises environmental concerns. During use, PVC materials present in pipes, cables, packaging films, flooring, or disposable products can gradually release additives or plasticizers (e.g., phthalates, Pb, Cd), contributing to soil and water pollution due to sun exposure or mechanical wear, as they generate microplastic fragments that persist in the environment and act as a means of transporting hazardous additives, while low recycling rates and inadequate disposal favor their accumulation (Henkel et al., 2022; Boyle et al., 2020) [7,8].

In light of this scenario, the modern industry is under growing pressure to operate according to sustainability criteria, driven both by consumers who prefer more eco-friendly options and by regulators who increasingly require companies to meet standards for resource efficiency and pollution reduction, while also needing to improve competitiveness and align with the goals of sustainable development. This rising environmental awareness, together with national and international policies, has spurred the development of tools to assess and mitigate the impact of chemical processes toward responsible and sustainable solutions. Among these standardized tools are the environmental impact assessment (EIA), which can reduce a project’s negative effects before it is implemented, and the life cycle assessment (LCA), which examines the material and energy balance from production to the final product [9].

In this context, one of the most effective strategies to reduce environmental impacts from the outset is integration of mass and energy within processes. Unlike traditional corrective methods, these strategies focus on pollution prevention during process design in line with the principles of industrial sustainability. El Halwagi has devised a structured approach that sets targets for waste reduction and energy consumption, making it possible to design cost-effective solutions that favor recovery and reuse of materials within the system and thus advance process integration [10].

To complement these approaches, tools such as the source sink diagram and mass exchange networks (MENs) help identify more precisely which streams can be recirculated directly, combined to meet composition limits, or subjected to specific pretreatment within the process, taking into account not only environmental effectiveness but also cost and operational feasibility [11].

Another fundamental element in the pursuit of a truly sustainable process is the proper management of water resources. This is especially critical in an industry like PVC production, which demands large volumes of water at various stages. Wastewater regeneration using membrane bioreactors is essential since these systems enable the complete degradation of polyvinyl alcohol (PVA) and, after an adaptation period, achieve the effective removal of chemical oxygen demand (COD) and biochemical oxygen demand within five days (${BOD}_{5}$). Thanks to this treatment, the resulting effluent not only meets quality requirements for water reuse in the process but also minimizes the risk of oxygen depletion in receiving bodies or in systems of internal recirculation [12]. These integrated strategies reduce water consumption, dependence on external water sources, and the volume of pollutant discharges, making a notable contribution to sustainability and reductions in environmental impact.

Currently, to quantify and objectively compare the impacts or benefits these improvements yield, one can use Computer-Aided Process Engineering (CAPE). This tool allows the simulation and optimization of material and energy balances and of operating conditions as a whole. It helps manage the complexity of streams, reactions, and energy, facilitating their analysis. It enables the exploration of different process configurations to see how they respond to changes in parameters and detect environmental issues before implementation [13].

In this way, the tool serves as the basis for algorithms such as WAR, which is used to calculate the potential environmental impact (PEI) of the flows of mass and energy in various processes. This takes into account four PEI indices and eight impact categories, four global and four toxicological, to produce a score that shows how sustainable each option under study is. Its graphical interface, WAR GUI, allows easy data entry, result visualization, and case comparison. It should be noted that it requires only data from the manufacturing stage and not from the entire life cycle of the product, which makes it a useful and practical tool for reducing waste and energy consumption [14].

WAR GUI has been employed in many academic studies, demonstrating great versatility in integrating with different types of analyses and simulators such as Aspen Plus or SuperPro Designer for various industrial processes, providing key information for decision-making. In the dairy industry, for example, Gomez Soto et al. (2020) combined the WAR algorithm with SuperPro Designer to evaluate not only the PEI of the process but also profitability indicators, allowing them to compare and identify the most efficient whey valorization route in economic and environmental terms [15]. In the work by Cassiani et al. (2018), it was used together with Excel to quantify the total PEI of the stages with the highest environmental burden and to assess how the choice of energy source affected the total PEI in agar production from Gracilaria [16]. In the field of advanced materials engineering, Arteaga Diaz et al. (2019) used WAR GUI to evaluate the production of magnetite nanoparticles by coprecipitation, concluding that the method generates low environmental impacts and is viable for industrial scaling [17].

In the biorefinery sector, Herrera Aristizábal et al. (2017) integrated simulation data into WAR GUI to calculate the PEI generated and emitted during the production of palm oil, biohydrogen, and palm kernel oil, identifying hydrogen generation as the stage contributing most to the PEI [18]. Similarly, González Delgado et al. (2017) studied a biohydrogen production process from African oil palm biomass via gasification, complementing process simulation with impact quantification across eight categories, which allowed comparisons of different energy and feedstock scenarios [19]. In the field of microalgae biofuels, González Delgado et al. (2022) also applied the WAR algorithm after simulating biodiesel production from the microalga Chlorella vulgaris in Aspen Plus. This enabled them to calculate the PEI generated and emitted per kilogram of biodiesel, showing that the process consumed impacts and thus represented a promising option in emerging biofuels [20].

Meramo et al. (2022) [21] used WAR GUI in the fermentation of cassava residues to produce acetone, butanol, and ethanol (ABE). For the environmental assessment, they selected the WAR algorithm and supplemented it with energy indicators (NER) and operational safety metrics, which made it possible to compare two valorization routes and propose safer and more sustainable configurations for residue utilization [21]. Even in pharmaceutical bioprocesses, González Delgado et al. (2022) evaluated influenza vaccine production in MDCK cells, where the software showed that the process consumed impacts and allowed them to determine which energy source most influenced environmental performance and suggest improvements to keep PEI low, identifying natural gas as the option with the least impact [22]. Finally, in the polymer field, Velásquez Barrios et al. applied this methodology to quality transitions in polypropylene production in Colombia, finding that adjusting changeover times between resin grades reduces plastic waste and potential environmental impact [23]. Monteiro et al. combined sustainability metrics based on WAR to compare six dimethyl carbonate synthesis routes, integrating economic criteria and ${CO}_{2}$ sequestration to select the most viable ecotechnologies [24].

Although studies have already employed computational methods to estimate the environmental impact of industrial processes, most previous research on PVC has considered only overall production without focusing on a specific stage or evaluating configurations that include strategies for structural process improvement. Likewise, important aspects such as mass integration, energy integration, or wastewater treatment are rarely taken into account, preventing a more complete perspective of the true environmental performance of these plants.

For example, González-Delgado et al. (2023) applied ASPEN Plus together with WAR GUI to quantify the environmental impact of PVC suspension polymerization but did not consider stream optimizations or energy-saving measures [25]. Later, Mendivil-Arrieta et al. (2025) carried out a pinch analysis to reduce thermal demand but did not include wastewater regeneration or a full life cycle assessment [26]. In another approach, Guardo-Ruiz et al. (2025) implemented a direct wastewater recycling system and evaluated its benefits using WEP indicators, although they did not apply the WAR method to quantify environmental impacts [27].

When examining other polymers, the pattern persisted. In 2025, Hu et al. performed a cradle-to-grave life cycle assessment on recycled polyethylene terephthalate (PET) flakes in Taiwan, and although they reported impact variations among three plants and recommended closed-loop washing and renewable energy, they did not integrate mass recovery, energy recovery, or water regeneration systems [28]. Similarly, Velásquez-Barrios et al. (2019) used the WAR algorithm in continuous polypropylene (PP) production to analyze how grade changeover strategies affected waste generation and total PEI [23], and Mannheim and Simenfavi (2020) conducted a life cycle assessment of injection molded polypropylene (PP) production, considering mass and water integration during production but not energy integration [29].

It is evident that no previous study simultaneously combines mass integration, energy integration, and wastewater regeneration in a single environmental assessment framework. In response to this gap, the present study proposes an environmental evaluation of PVC suspension polymerization that includes mass recovery, energy recovery, and wastewater regeneration under a zero-discharge approach. When implemented together, these strategies have the potential to significantly reduce environmental burdens related to resource consumption and waste generation. To quantify clearly and objectively the environmental benefits of these improvements, WAR GUI will be used so that potential impacts can be analyzed in detail across different scenarios, thereby supporting decisions aimed at cleaner and more sustainable production [14]. Table 1 compares the various studies on environmental analysis in PVC and other polymer processes, highlighting that this work is the first to integrate mass integration, energy integration, and wastewater treatment using WAR GUI for environmental evaluation.

## 2. Materials and Methods

### 2.1. Environmental Assessment Using WAR Algorithm

To quantify the environmental performance of our PVC suspension process, we used the Waste Reduction Algorithm (WAR), a systematic methodology to quantify the Potential Environmental Impact (PEI) generated by a chemical process. WAR measures the material flows entering and leaving the system, as well as the impacts related to the product’s energy consumption during its manufacture [14].

Unlike a Life Cycle Analysis (LCA), which encompasses raw material extraction, distribution, use, disposal, and recycling, WAR focuses solely on the manufacturing stage. This focus allows the calculation of both the impact released to the environment and the net PEI generated within the system, results that cannot always be obtained with a basic life cycle analysis [18].

For these reasons, and given that the objectives of this study focus on evaluating and optimizing these operational stages and process scenarios, the WAR algorithm was chosen; its focus on manufacturing and its ability to quantify both the PEI transferred to the environment and the net PEI generated internally allow for accurate comparisons of the environmental footprint of different process scenarios.

The algorithm is based on a mass and energy balance that allows tracking how potential environmental impacts are generated or transferred within the system [14]. Within this framework, WAR defines two key index classes. The first measures the PEI leaving the system as emissions, waste, or losses and reflects the environmental impact emitted by the process to its surroundings, allowing assessments of the process’s capacity to produce goods with lower PEI emissions. This quantity can be expressed as a rate per unit time (to estimate the total PEI leaving the system each hour or day) and as a rate per unit mass (to compare the environmental efficiency of different processes based on PEI emitted per unit of product). These impacts are calculated using Equations (1) and (2) [30]:

(1) $${\;i}_{out}^{\left(t\right)}=i_{out}^{\left(cp\right)}+i_{out}^{\left(ep\right)}+i_{we}^{\left(cp\right)}+i_{we}^{\left(ep\right)}=\sum_{i}^{cp}{Mj}^{out}\sum_{k}^{cp}X_{kj}\varphi_{k}+\sum_{j}^{ep-g}{Mj}^{out}\sum_{j}^{ep-g}X_{kj}\varphi_{k}$$

(2) $$i_{out}^{\left(t\right)}=\frac{i_{out}^{\left(cp\right)}+i_{out}^{\left(ep\right)}+i_{we}^{\left(cp\right)}+i_{we}^{\left(ep\right)}}{\sum_{P}P_{P}}=\frac{\sum_{i}^{cp}{Mj}^{out}\sum_{k}^{cp}X_{kj}\varphi_{k}+\sum_{j}^{ep-g}{Mj}^{out}\sum_{k}^{ep-g}X_{kj}\varphi_{k}}{\sum_{P}P_{P}}$$

The second index measures the potential environmental impact (PEI) generated within the system: that is, the impact produced because of chemical reactions or energy consumption during production. This index helps understand the internal environmental efficiency of the process, since it shows the rate at which the process itself generates new impacts. Similarly to the output impacts, it can be expressed as a rate per unit of time to determine how quickly impacts are generated or as a rate per unit of product mass to enable comparison with other processes. The lower these values, the more environmentally efficient the system will be. These calculations correspond to Equations (3) and (4) [30]:

(3) $$\;\;i_{gen}^{\left(t\right)}=i_{out}^{\left(cp\right)}-i_{in}^{\left(cp\right)}+i_{out}^{\left(ep\right)}-i_{in}^{\left(ep\right)}+i_{we}^{\left(cp\right)}+i_{we}^{\left(ep\right)}\;\;=\sum_{i}^{cp}{Mj}^{out}\sum_{k}^{cp}X_{kj}\varphi_{k}-\sum_{j}^{cp}{Mj}^{\left(in\right)}\sum_{k}^{cp}X_{kj}\varphi_{k}+\sum_{j}^{ep-g}{Mj}^{\left(out\right)}\sum_{k}^{ep-g}X_{kj}\varphi_{k}$$

(4) $$i_{gen}^{\left(t\right)}=\frac{i_{out}^{\left(cp\right)}-i_{in}^{\left(cp\right)}+i_{out}^{\left(ep\right)}-i_{in}^{\left(ep\right)}+i_{we}^{\left(cp\right)}+i_{we}^{\left(ep\right)}}{\sum_{P}P_{P}}\;\;\;\;\;=\frac{\sum_{j}^{cp}{Mj}^{out}\sum_{k}^{cp}X_{kj}\varphi_{k}-\sum_{j}^{cp}{Mj}^{\left(in\right)}\sum_{k}^{cp}X_{kj}\varphi_{k}+\sum_{j}^{ep-g}{Mj}^{\left(out\right)}\sum_{k}^{ep-g}X_{kj}\varphi_{k}\;}{\sum_{P}P_{P}}$$

In these equations, some specific variables appear, such as $i_{out}(cp)$ and $i_{in}(cp)$, which represent the output and input PEI rates related to chemical interactions within the process; $i_{out}(ep)$ and $i_{in}(ep)$, which relate to PEI rates linked to energy generation processes within the system; and $i_{we}(ep)$ and $i_{we}(cp)$, which represent the energy wasted in the system, reflecting the impacts generated by the release of unused energy during chemical reactions and energy generation.

Moreover, the mass flow rates of the system streams are represented as $M_{j}(in)$ and $M_{j}(out)$, which simply denote the amount of material entering and leaving stream (*j*); the mass fraction of each component *k* in stream *j* is denoted $X_{k}$, and the global potential of chemical substance *k* is $\psi_{K}$. Finally, $P_{p}$ refers to the mass flow rate of product *p* [31].

To estimate the impacts of substances present in the process, the WAR algorithm considers eight categories divided into two groups: the first addresses toxicological effects on organisms, and the second covers contributions to atmospheric deterioration.

Within the first group related to toxicological effects, the human toxicity potential by ingestion (HTPI) is included. This metric measures the hazard of substances that may be in a liquid or solid state at standard temperature (273.15 K) and pressure (1 atm). Its estimation uses the median lethal dose for rats via the oral route (LD_50_): in other words, the amount of substance that would cause the death of 50 percent of a rat population by oral ingestion. HTPI is defined by Equation (5) [31,32]:

(5) $$HTPI:\;\frac{1}{{LD}_{50}}$$

As shown in Equation (5), to maintain coherence in the impact evaluation scale, the algorithm inverts the ${LD}_{50}$ value so that a substance with higher toxicity (that is, with a lower ${LD}_{50}$) generates a higher HTPI value. In this way, it is possible to prioritize the most hazardous substances from the oral toxicity perspective. This parameter is quantified in units of mg of chemical per kg of rat [32].

Another metric in this group is the human toxicity potential by inhalation or dermal exposure, HTPE, which focuses on the effects caused by gaseous compounds when inhaled or in contact with skin. This value is estimated using the eight-hour time-weighted average exposure limit TLV as established by regulatory bodies such as OSHA, ACGIH, and NIOSH. TLV units are expressed in milligrams per cubic meter for these substances at standard temperature 273 K and pressure 1 atm [32]. The equation representing the calculation of this indicator is Equation (6):

(6) $$HTPE:\;\frac{1}{TLV}$$

As with HTPI, the TLV value is inverted so that a lower TLV yields a higher potential impact. It is worth noting that certain substances that exist in more than one physical state under standard operating conditions may present both HTPI and HTPE. Just as in the case of HTPI, we see that its value is inverted in order to preserve the logic of the index: The lower the TLV, the greater the potential impact. It is important to note that certain substances, which exist in more than one physical state under standard operating conditions, may exhibit both HTPI and HTPE.

The next metric in the toxicological category is the aquatic toxicity potential (ATP). This metric evaluates the risks that substances pose to freshwater organisms. Fathead minnows are typically used for this assessment due to the wide availability of data and their acceptance as a universal indicator. The base measure is the median lethal concentration ${LC}_{50}$, which corresponds to the exposure level in milligrams per liter that causes the death of 50 percent of the fish population over a period normally set at 96 h. Equation (7) shows how this is quantified [31,32]:

(7) $$ATP:\;\frac{1}{{LC}_{50}}$$

The fourth metric in this toxicological group is the terrestrial toxicity potential (TTP). This estimates the hazard to terrestrial organisms using rats as the experimental model owing to their acceptance as representative and the wide availability of data. Its calculation is similar to that of HTPI but is directed at the terrestrial environment, as shown in Equation (8) [31,32]:

(8) $$TTP=\;\frac{1}{{LD}_{50}}$$

The other four categories considered, as previously mentioned, are related to atmospheric effects. Two of them correspond to global-scale impacts, while the other two are of local or regional nature (PCOP and AP). Among the global impacts, the first is the ozone depletion potential (ODP), which assesses a substance’s ability to contribute to the destruction of the stratospheric ozone layer. This is determined by comparing the rate at which a unit mass of the chemical reacts with ozone to form molecular oxygen relative to the rate for CFC-11 (trichlorofluoromethane), which is used as the reference substance [31,32]. Equation (9) shows how it is calculated:

(9) $$ODP=\frac{\delta [O_{3}{]}_{1}}{\delta \left[O_{3}\right]FCKW-11}m_{1}$$

Compounds with significant ODP typically contain chlorine or bromine atoms, which, upon degradation, release radicals capable of destroying ozone molecules. The ODP is expressed in kilograms equivalent of CFC-11, and its calculation considers both the emitted mass of the gas and its relative depletion potential [31].

The second global impact category is the global warming potential (GWP), which quantifies a substance’s contribution to the greenhouse effect by comparing it to carbon dioxide (${CO}_{2})$. It is more clearly defined by comparing the amount of infrared radiation that a unit mass of the chemical can absorb over a 100-year period to the amount absorbed by an equivalent mass of (${CO}_{2})$ in the same time frame [31,32]. This metric takes into account the substance’s degradation in the atmosphere and its evolution over time. Equation (10) shows how it is calculated:

(10) $$GWP=\frac{\int_{0}^{t}a_{1}c_{1}\left(t\right)dt}{\int_{0}^{t}a_{CO_{2}}c_{{CO}_{2}}\left(t\right)dt}m_{1}$$

The GWP result is expressed in kilograms of ${CO}_{2}$ equivalent per kilogram of emitted substance, allowing identification of the gases with the highest greenhouse effect and their influence on climate.

Now, regarding the regionally focused categories, the acidification potential (AP) is included, also known as acid rain potential, which—as its name suggests—represents a substance’s capacity to contribute to the formation of acid rain. It is determined by comparing the rate of $H^{+}$ release promoted by a chemical to the rate of $H^{+}$ release in the atmosphere promoted by ${SO}_{2}$, which is used as the standard reference [31,32]. It is calculated using Equation (11):

(11) $$AP=\frac{\frac{V_{i}}{M_{i}}}{\frac{V_{{SO}_{2}}}{M_{{SO}_{2}}}}m_{1}$$

The release of $H^{+}$ is responsible for the acidification of water bodies and soils, negatively affecting ecosystems and material structures. The AP value is expressed in kilograms of ${SO}_{2}$ equivalent per kilogram of emitted substance.

Lastly, the photochemical oxidation potential (PCOP), also referred to as smog formation potential (SFP), is determined by comparing the rate at which a chemical compound reacts with hydroxyl radicals (OH) in the atmosphere to the reaction rate of ethylene with the same radicals [32]. In other words, it measures the ability of volatile organic compounds (VOCs) to form ozone in the troposphere through reactions with these radicals. The result is expressed in kilograms of ethylene equivalent emitted. The corresponding calculation is shown in Equation (12):

(12) $$PCOP=\frac{\frac{a_{i}}{b_{i\;}(t)}}{\frac{a_{C_{2}H_{4}}}{b_{C_{2}H_{4}}\;(t)}}m_{1}$$

where $a_{i}$ is the change in ozone concentration due to a change in the emission of a specific volatile organic compound *i*, $a_{C_{2}H_{4}}$ refers to the same change with respect to ethylene emission, $b_{i\;}(t)$ is the integrated emission of compound *i* over time *t*, $b_{C_{2}H_{4}}$ (t) refers to the same condition for ethylene, and m_i_ is the mass (kg) of the emitted volatile organic compound [31,32].

### 2.2. Process Description

With the aim of improving the suspension PVC production process and making it more sustainable, improvement strategies such as wastewater reuse and mass and energy integration have been implemented. To define operating conditions and collect data from full-scale industrial operations, the simulation model developed by Aguilar-Vásquez et al. [33] was used as a basis; this model was built from operational data of real plants obtained under confidentiality agreements and from the specialized body of literature. The model corresponds to a reference plant with a nominal production of 467,436.3 tonnes per year (t/year) of PVC; on this basis, the mass and energy flows used in the environmental analysis were calculated.

**Figure 1:**
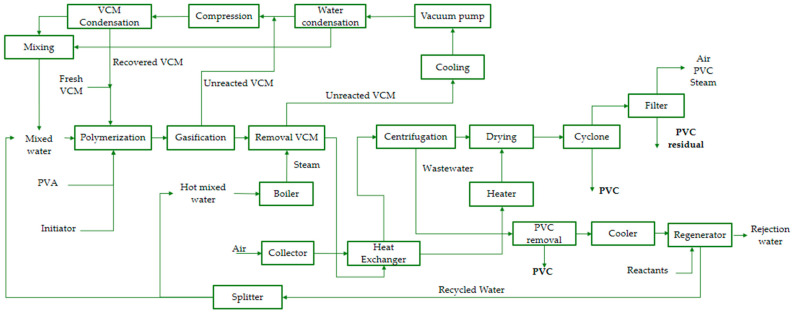
Process flow diagram for mass-integrated suspension PVC production with wastewater regeneration.

In this context, the functional unit used in this study is 1 ton of PVC produced (PVC resin). All key indicators (PEI generated and PEI produced) are presented by this FU (PEI/ton of product), and in addition, the rates per unit of time (PEI/day) are shown when the comparison requires incorporating the capacity or speed of impact generation of the process. Figure 1 shows the process flow diagram:

The process begins when the reactor is fed with a mixture of fresh and recycled vinyl chloride monomer (VCM) in an 80% fresh to 20% recycled ratio. The reaction is carried out in an aqueous suspension using polyvinyl alcohol (PVA) as a stabilizer and an initiator (3-hydroxy-1,1-dimethylbutan-2-yl-2-methylheptane). Reaction conditions are controlled near 70 °C and 10 kgf/cm^2^ (≈9.81 bar), and continuous cooling is applied to offset the exotherm. This stage reaches approximately 85% conversion, producing a slurry of PVC particles in water, together with a residual initiator, PVA, and unreacted monomer. The stream leaving the reactor is handled at roughly 3.5 kgf/cm^2^ (≈3.43 bar) and about 70 °C.

To separate the unreacted monomer, two sequential operations are performed. In the first stage, the slurry is sent to a flash vessel where the pressure is reduced to 1.8 kgf/cm^2^ (≈1.77 bar), volatilizing approximately 95% of the free monomer.

The vapor phase from the flash carries notable quantities of water (about 18 t/day) and small traces of PVA and initiator (<1% each), and it is routed to cooling, compression, and condensation units to recover and recycle VCM.

In the second stage, the liquid-phase purge stream enters a stripping column where high-pressure steam (generated at 14 kgf/cm^2^ (≈13.73 bar) and 225 °C) strips the remaining volatile monomer until the residual VCM in the bottom product is below 1 ppm [34,35]. The recovered monomer is condensed, compressed, and returned to the process as a liquid at approximately 3.5 kgf/cm^2^ (≈3.43 bar) and 8 °C. Steam demand for the column is supplied by a boiler with a capacity of 20 t/h.

After this desorption stage, the polymer-laden slurry passes through a shell-and-tube heat exchanger, where thermal energy is transferred to the air used in the drying stage. This preheats the air and reduces the fuel demand for the burner [35].

Then, the slurry contains a high water fraction (about 70% by mass) and is first dewatered in a centrifuge running at around 1800 rpm, which typically removes about 75% of the suspension water [33]. The resulting aqueous purge stream contains some polymer fines together with most of the removed PVA and residual initiator; the concentrated paste (≈25% moisture) proceeds to drying.

This paste is sent to a dryer, where a hot air stream at 250 °C reduces the moisture to 0.01%. Once the material is dried, it is separated from the air stream in a cyclone, and the finer particles are retained in a bag filter, achieving an operational efficiency of 99%. The dryer operation is controlled so that the resin bulk temperature remains near 70 °C, avoiding thermal degradation. Dried material is separated from the gas stream in a downstream cyclone operating essentially at atmospheric conditions (1.03 kgf/cm^2^, ≈1.01 bar) [33]. The cyclone collects most of the solid product at the bottom, while the overflow gas stream contains a small fraction of the powder (about 0.2% of the total polymer produced); these entrained fines are captured in a bag filter with a nominal removal efficiency of ~99%.

The wastewater generated (primarily during centrifugation) is directed to a regeneration system combining physico-chemical and biological treatment. In the physico-chemical stage, coagulation, flocculation, and clarification are applied using an aluminum-based coagulant, achieving a 99% removal efficiency. This stream is then cooled to 35 °C before entering two reactors, an anaerobic reactor and an aerobic reactor, designed for PVA degradation; they receive potassium oxide and air to facilitate biodegradation [34]. Finally, the treated water undergoes a reverse osmosis unit, simulated in Aspen Plus, where remaining impurities are removed and the regenerated water is separated from the reject stream; the reclaimed water is split and reused in the polymerization feed and in the boiler, closing the water loop and improving resource efficiency.

The analysis presented corresponds to a gate-to-gate approach, including direct inputs to the plant such as raw materials introduced into the system (fresh and recycled PVC, stabilizers, initiators, and additives) and utilities consumed in the factory (steam generated in boilers, grid electricity, and fuel for burners). Outputs are considered to be the final product (PVC), waste streams (liquid effluents from treatment, gaseous emissions from combustion, and particulate emissions from the drying line), and solid process waste.

Off-site stages (raw material extraction and production, etc.) are excluded unless explicitly indicated. Recirculated streams within the plant, e.g., recovered VCM or reused reclaimed water, are modeled as internal flows and are not counted as additional external inputs. Losses and streams directed to treatment/management are recorded as outputs for the calculation of output PEI (TPLS).

All operating parameters and the mass and energy allocations used for the environmental (WAR) analysis are documented in Aguilar-Vásquez et al. (2025) [33], which served as the basis for the case definition.

### 2.3. Environmental Assessment Using Computer-Aided Process Engineering

**Figure 2:**
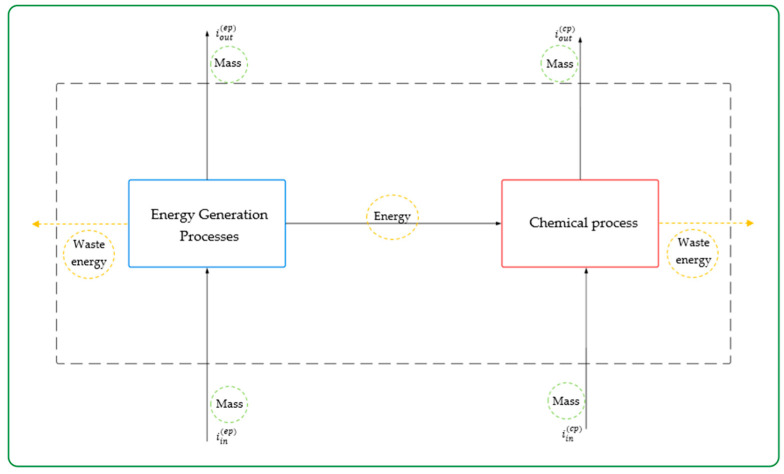
Material and energy balance and environmental impact of a chemical process.

An environmental assessment of the suspension PVC production process was carried out using the WAR GUI software v. 1.0.17, based on the Waste Reduction Algorithm (WAR). This tool was developed by the U.S. Environmental Protection Agency (EPA) in order to evaluate and minimize environmental impacts. To determine the potential environmental impacts (PEIs), Equations (1)–(4) were used, combining the material balance with the energy balance of the system (Figure 2), and Equations (5)–(12) were applied to quantify the specific contributions in the atmospheric and toxicological categories based on the composition and properties of each stream’s substances.

In order to compare different flow configurations and energy consumption, bar charts were generated for four practical cases. For clarity, these cases are incremental and were constructed sequentially to show the contribution of each group of flows to the total PEI. All cases are referenced to the functional unit of 1 t of PVC produced. Case 1 considered only the impacts derived from waste streams leaving the plant (effluents, gaseous emissions, and solid residues); Case 2 added the effects of the product (the PEI attributed to the product output) and waste flows, without accounting for energy consumption; Case 3 included both waste impacts and the energy contribution (fuel for boilers/vapor generation and electricity from the grid); finally, Case 4 accounted for product flow impacts, energy consumption, and waste impacts, thus providing a comprehensive view of environmental performance. This case was used to analyze both the PEI generated within the system and the PEI transferred to the environment across individual atmospheric and toxicological categories.

This classification made it possible to evaluate each system component sequentially, so with these four cases, the total impact figure was constructed. To deepen the analysis of the influence of energy consumption, Case 3 was employed to break down the contributions and obtain a more complete view of environmental performance. Daily PEI rates were also calculated for each process subsection, considering only waste and energy consumed.

To contextualize the WAR graphical user interface indicators, the results of this study were compared with the conventional study on PVC in suspension by González-Delgado et al. (2023) [25], using the same WAR categories and the same system boundaries (manufacturing). The comparisons are made directly on the values reported in PEI/day, focusing on the effect of water integration and regeneration.

## 3. Results

**Figure 3:**
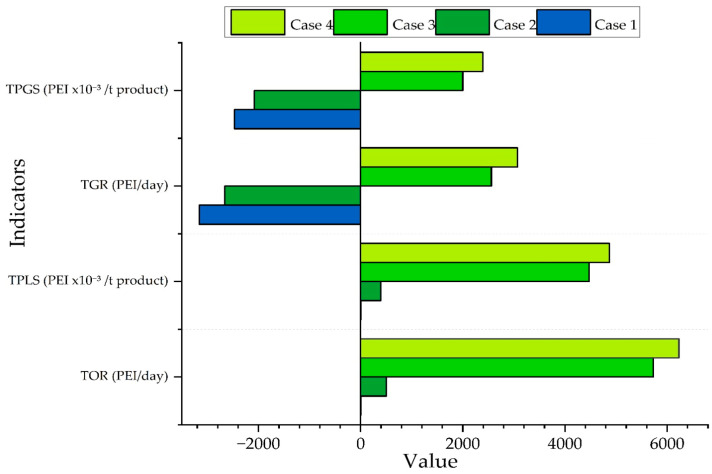
Total PEI generated and output of the PVC suspension process with integration for the four incremental cases.

As mentioned above, the four cases are incremental. Case 1 considers only the impacts derived from waste; Case 2 adds product flows while continuing to exclude energy consumption; Case 3 also includes the contribution of energy; Case 4 incorporates product flows, energy consumption, and waste. Figure 3 shows the total potential environmental impact (PEI) generated and the total PEI output per ton of product per day for the four evaluated cases. The generated PEI is quantified as the total generation rate (TGR, in PEI/day), while the discharged PEI is expressed as the total output rate (TOR, in PEI/day). To be able to compare on a mass basis, we also present these same flows per ton of product, reporting the total PEI generated per mass of product (TPGS, in PEI/t product) and the total PEI leaving the system per mass of product (TPLS, in PEI/t product).

In Case 1, the total PEI generated is −3160 PEI/day and −2.47 PEI per ton of product, while the output remains very low at 2.46 PEI/day and 0.00192 PEI per ton of product. This behavior primarily reflects a net consumption of impacts due to the conversion of the vinyl chloride monomer (VCM) into Poly(vinyl chloride) (PVC) and the recirculation of the unreacted monomer. VCM has an ${LD}_{50}$ of 500 mg/kg and an aquatic ${LC}_{50}\;$ of 26 mg/L, whereas PVC has an ${LD}_{50}\;$ of 2000 mg/kg and an aquatic (${LC}_{50})\;$ of 100 mg/L. Furthermore, coagulation with aluminum sulfate (${LD}_{50}\;=\;1930\;mg/kg;\;{LC}_{50}\;=\;33.9\;mg/L$) and biological treatment reduce the toxicity load in the outlet streams, so only the concentrated reject and sludge enter the wastewater system.

In Case 2, which also considers the product flow, the generated PEI changes to −2660 PEI/day and 2.08 PEI per ton of product, while the output rises to 505 PEI/day and 0.394 PEI per ton of product. This increase is due to the inclusion of the impact associated with the produced PVC, along with its traces of polyvinyl alcohol (${LD}_{50}$: 2000 mg/kg; ${LC}_{50}$: 100 mg/kg) and initiator (${LD}_{50}$: 300 mg/kg; ${LC}_{50}$: 1 mg/L), plus the rejected water and sludge that raise the output load.

In Case 3, which considers energy consumption, the generated PEI becomes 2560 PEI/day and 2 PEI per ton of product. The outlet impacts increase to 5730 PEI/day and 4.47 PEI per ton of product. This is mainly attributable to the contribution of high-pressure steam, the heat exchanger, and the reverse-osmosis stage, for which its greenhouse-gas emissions are linked to their production and operation. In systems fueled by natural gas, carbon dioxide accounts for more than 99% of air emissions, while methane makes up most of the remainder due to leaks during combustion and fuel handling [36,37]. Each kilogram of steam generated increases the GWP through ${CO}_{2}$ emissions (GWP = 1) and methane (${CH}_{4}$) emissions (GWP = 23) [38], outweighing the toxicological advantage gained from VCM recirculation and prior water treatment.

Finally, in Case 4, which integrates waste, product flow, and energy consumption, the total generated PEI is 3070 PEI/day and 2.39 PEI per ton of product, while the PEI output reaches its maximum values of 6230 PEI/day and 4.87 PEI per ton of product. This increase is due to the reject stream from the reverse-osmosis stage, containing substances such as sodium hypochlorite (${LD}_{50}$ = 8900 mg/kg; ${LC}_{50}$: 26 mg/L) and potassium phosphate (${LD}_{50}$ = 2410 mg/kg; ${LC}_{50}$ = 83 mg/L), together with the contributions from energy and product flows, resulting in a higher output profile.

The progression of the output values summarizes the contribution of each process stream: first, mass integration and almost complete monomer recirculation keep the initial impact at minimal levels; adding the product flow increases it slightly; with energy demand and the reject streams from the reverse-osmosis and sludge stages, the largest share of the output footprint is obtained.

The results show that the integrated system with wastewater regeneration delivers a notable improvement in environmental performance compared to other studies. In Case 1, an output of 2.46 PEI/day is achieved, representing a 62% reduction compared to the 6.5 PEI/day reported by González-Delgado et al. (2023) for conventionally produced suspension PVC [25]. This improvement can be attributed primarily to the biological treatment and aluminum sulfate coagulation stages, which minimize the toxicity of the liquid effluents. The high efficiency in converting VCM to PVC also enhances this system, as reflected by a more negative generated PEI of −3160 PEI/day versus −2800 PEI/day in the baseline study [25], indicating a greater capacity to transform toxic substances into the desired product.

In Case 2, the regenerated model shows an increase in PEI output (505 PEI/day) compared to 457 PEI/day in the referenced study, corresponding to a 10.5% rise. However, the regenerative system maintains a significant advantage: the generated PEI of −2660 PEI/day represents an 11% improvement in net impact reduction compared to −2400 PEI/day in the baseline study [25], confirming greater efficiency in transforming the process’s toxic substances.

The most significant differences appear in Cases 3 and 4, where the added value of the regeneration system becomes even clearer. Although the regenerated system uses 12% more energy (5184 GJ/day versus 4625 GJ/day [25]), the overall system still reduces PEI output. In Case 3, there is a 2.6% reduction, with 5730 PEI/day versus 5890 PEI/day; in Case 4, there is a 1.7% decrease compared to the baseline value of 6340 PEI/day [25]. This apparent contradiction—higher energy consumption but lower environmental impact—is explained by the regeneration technologies’ ability to concentrate dispersed pollutants into manageable loads, which partially offset the energy-related emissions and reduce the total output PEI [39].

By comparing with other industrial chemical processes, such as the production of n-butyl acetate, it was found that the conventional process yields an output of 9744 PEI/day and an energy consumption of 436.14 GJ/day [40]. In contrast, Case 4 of this study exhibits an output of only 6230 PEI/day, despite its higher energy demand, representing a 36% reduction in effluent emissions. When compared with reactive distillation of acetate, that process shows an output PEI of 1344 PEI/day and a net PEI generation of −33,600 PEI/day, reflecting very high efficiency in impact transformation and neutralization [40].

In this context, the different case studies evaluated demonstrate that the integrated system with wastewater regeneration achieves a competitive environmental performance. Although Case 4 presents a relatively high output load (approximately 4.6 times greater than reactive distillation), this difference is justified by the greater toxicological complexity of the PVC process, which involves highly hazardous and persistent compounds such as VCM, sodium hypochlorite, and PVA. However, the implemented technologies (reverse osmosis, coagulation, and biological treatment) provide effective treatment by containing and concentrating most of that toxicity in specific streams, thereby reducing the environmental impact. As a result, this system proves to be more robust environmentally; even with higher energy demand, it maintains low or comparable PEI output levels to less demanding processes, demonstrating both the technical and environmental feasibility of the selected approach.

To deepen the analysis, Table 2 breaks down the contribution of each process unit to the PEI associated exclusively with reject streams. From the WAR data, it was identified that the greatest contribution to environmental impact occurs in the wastewater regeneration stage, with a value of 0.858 PEI/day, equivalent to 95% of the total. This outcome is due to the nature of the reject stream generated in the reverse-osmosis unit, which acts as the final concentrator of substances that resist both physico-chemical and biological treatment. Although traces of substances such as PVA, initiator, and VCM-derived compounds appear in minimal proportions, because they are not recirculated into the system, they accumulate in this residual stream, making it the primary exit point for contaminants.

By contrast, the drying stage makes a very small contribution at 0.05 PEI/day (0.05%). This impact is attributed mainly to ultrafine PVC particles entrained by the air flow and retained in the filtration system. While this value is small relative to the total processed, it is measurable due to the toxic load of PVC when not properly managed. This output is directly associated with the final equipment in the drying line (cyclone and bag filter), which—even with 99% efficiency—cannot achieve absolute retention.

In contrast, the polymerization, purification, and VCM recovery stages show no directly measurable environmental impacts, as they do not generate reject streams. This is mainly due to the high degree of process integration, namely intermediate stream recirculation, closed-loop water management (which confines liquid emissions to a controlled stream), and thermal recovery in the heat exchanger.

In summary, the PEI output value obtained in this process highlights the efficiency of its application. The low generation of toxic waste, combined with recovery strategies, maintains a minimal environmental profile—even when operating with toxically sensitive raw materials and at large volumes. This aligns with studies in wastewater treatment plants that also integrate water and energy recovery, which report significant reductions in environmental impact approaching net-zero [41].

**Figure 4:**
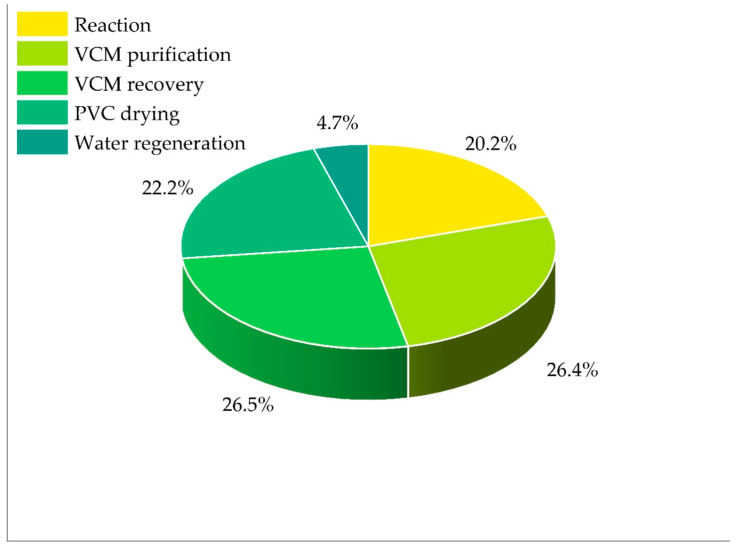
Contribution of PEI output rate per process section (Case 3: diagnostic case for energy contribution).

Figure 4 shows the contribution of each stage to the PEI output of the process, taking into account energy consumption (Case 3). The VCM recovery and purification stages contribute the most to the total impact at 26.53% and 26.35% respectively. This is due to the use of high-pressure steam $\left(14\;kgf/{cm}^{2}\;at\;225\;{}^{\circ}C\right)$ required for monomer desorption and purification, as well as the subsequent compression, condensation, and monomer recirculation steps. In addition, these stages involve handling highly toxic substances, such as VCM itself and traces of initiator, which increase the final impacts.

The drying stage also makes a significant contribution at 22.16% of the total PEI, and this is related to the need to heat air to 250 °C to remove residual moisture from the polymer. Although the air is partially preheated via a heat exchanger, the operation remains a considerable source of energy consumption and emissions. Ultrafine PVC particles entrained in the air further contribute to this category, despite the filtration system’s 99% efficiency.

The reaction stage contributes 20.24% of the PEI output. Although no additional heat is required thanks to the reaction’s exothermicity, the use of polyvinyl alcohol (PVA) as a stabilizer and highly toxic initiators adds to the output value. Moreover, the cooling system used to maintain the temperature at 70 °C consumes energy through compressors and refrigerants, albeit to a lesser extent than other stages.

Finally, the regeneration stage has the smallest contribution at 4.71% thanks to the efficiency of the physico-chemical and biological treatments in containing residual toxicity. Although this unit handles a large fraction of the process’s liquid waste, its impact remains low due to the zero-discharge approach and internal reuse, especially toward the boiler and reactor.

### 3.1. Toxicological Impacts of the PVC Suspension Production Process

**Figure 5:**
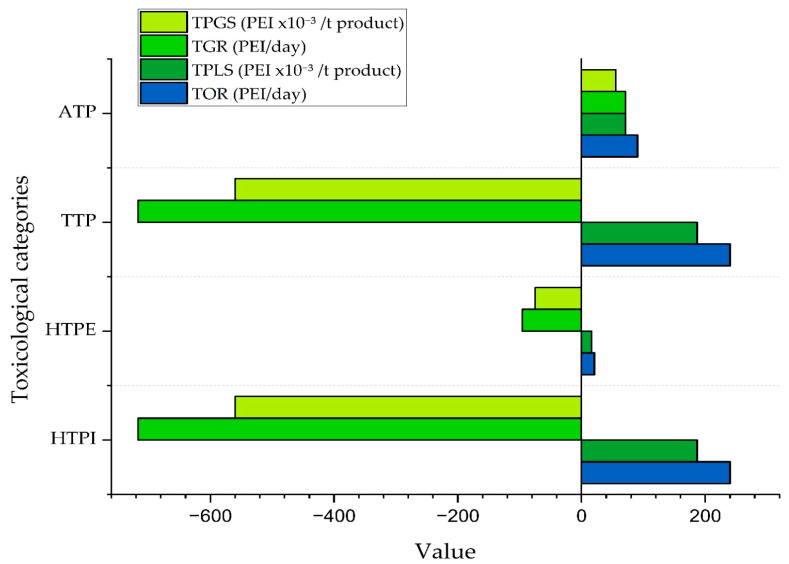
Toxicological impacts of the PVC suspension production process, including waste streams, product streams, and energy consumption (Case 4).

Figure 5 presents the toxicological impact rates generated and resulting from the suspension PVC production process, grouped into human ingestion toxicity (HTPI), human environmental exposure toxicity (HTPE), terrestrial toxicity potential (TTP), and aquatic toxicity potential (ATP). The results indicate that the first three categories achieve significant reductions in environmental impact, with negative generation rates of −717.00 PEI/day (HTPI), −95.80 PEI/day (HTPE), and −717.00 PEI/day (TTP). This outcome is directly related to the high reactor conversion (85%) and the effective recovery of unreacted VCM, which prevents its release as waste.

In contrast, the ATP category shows a positive generation rate of 90.70 PEI per day, indicating that some substances present in the liquid waste or retained in the final product contribute to the toxic load in aquatic environments. Notably, PVA and the initiator, although at low concentrations, have low ${LC}_{50}$ values. Moreover, the stream from the bottom of the cyclone may contain fine solids that elevate this category’s impact by behaving like microplastics. Studies have shown that such particles can leach toxic additives such as phthalates into water over long periods, increasing system toxicity and transporting persistent compounds into ecosystems [7].

Figure 5 also shows that all categories have positive PEI outputs, which means that despite significant internal reductions, toxic substances still exit into the environment. The HTPI and TTP categories both register high output values of 240 PEI per day, reflecting the influence of the final product stream, which represents the largest flow rate in the system. The HTPE category, by comparison, has a lower value of 20.90 PEI per day, attributed to emissions of ultrafine PVC particles that become airborne during handling of the dry product. The direct impact of VCM is limited because it is extensively recirculated within the process. Although VCM’s threshold limit value of 2 mg per cubic meter is lower than that of PVC, its output impact remains minor [25]. Regarding ATP, the value of 90.70 PEI per day can be attributed to traces of PVA, initiators, or ultrafine solid particles in the liquid streams or in the cyclone underflow, which, even at low concentrations, have a high toxicity potential for aquatic organisms [8,42].

Analyzing the output impacts per unit mass of product, all categories exhibit low values: 0.19 PEI per ton of product for HTPI and TTP, 0.02 PEI per ton for HTPE, and 0.07 PEI per tonne for ATP. The identical HTPI and TTP values arise because both categories are determined by the same substance, PVC, which has a similar impact coefficient in these two toxicological indicators, implying that PVC drives those environmental effects.

With respect to HTPE, the impact difference is minimal because when expressed per ton of product, only ultrafine PVC particles that escape the drying stage remain in the output stream, and any residual gaseous emissions are negligible once processed internally. ATP remains positive, indicating that substances capable of affecting aquatic organisms still persist in the system. As noted earlier, this may be due to traces of PVA and initiator that, even at low concentrations, have low ${LC}_{50}$ values, making them highly toxic to aquatic life. In addition, very small particles in the liquid streams or cyclone underflow may promote the release of hazardous additives into the aquatic environment.

Regarding the contribution of output impacts per mass of product, the values are −0.56 PEI per ton of product for HTPI, −0.07 PEI per ton for HTPE, −0.56 PEI per ton for TTP, and 0.06 PEI per ton for ATP. These figures reflect the efficiency of the regeneration stage, showing reductions in impacts associated with toxic substances. They indicate that this operation effectively removes compounds harmful to human health and the environment. Finally, ATP continues to show a slight positive impact, which may be associated with the energy consumption of the regeneration stage and its small thermal load on the system, demonstrating that although there are significant environmental benefits, there remains room for improvement in energy performance.

### 3.2. Atmospheric Impacts of PVC Suspension Production Process

The ozone depletion potential (ODP) category shows identical generated and output values (0.00188 PEI per day), indicating that the compounds responsible for this impact are neither retained nor transformed by the system. Although this value is very low compared to other impact categories, its persistence suggests that substances such as VCM and traces of initiator may contain small amounts of chlorine, which can be released and contribute to stratospheric ozone depletion. While the process includes VCM recirculation and recovery, which significantly reduces these emissions, there is no dedicated stage designed to treat or neutralize ODP-relevant compounds, explaining why this impact remains in the output. Furthermore, using natural gas as an energy source, although producing fewer impacts than other fossil fuels, can still release small quantities of halogenated compounds.

In contrast, the photochemical oxidation potential (PCOP) results are favorable. The generated impact value is −1110 PEI per day, demonstrating a significant reduction in substances capable of forming tropospheric ozone. This can be attributed to the high efficiency of converting VCM into PVC, intensive VCM recirculation, and the wastewater treatment stages. In particular, combining physico-chemical treatment with both aerobic and anaerobic biodegradation of PVA removes volatile organic compounds, preventing their release into the environment.

The PCOP output value is 0.37 PEI per day and zero when expressed per unit of product, confirming the system’s efficiency at avoiding significant atmospheric emissions. This low result also stems from the effective filtration performance in the drying stage, where the cyclone and bag filter retain almost all PVC particles carried in the hot air. Nonetheless, small fractions of PVA or initiator might still escape in gaseous or liquid form, which could explain the remaining impact. In summary, the process acts as a net consumer of PCOP, efficiently preventing its release, whereas the ODP remains unchanged due to the lack of a specific treatment for chlorine-containing compounds, yet overall, the system achieves a very favorable atmospheric performance with respect to tropospheric ozone formation.

**Figure 6:**
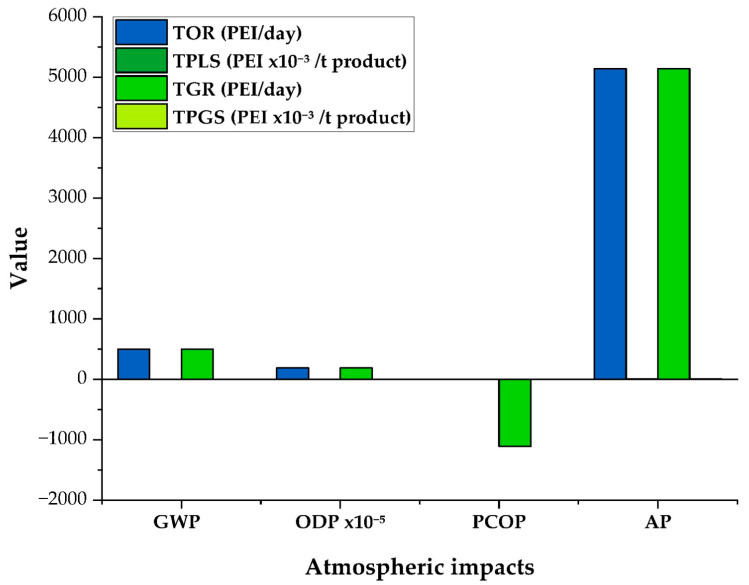
Atmospheric impacts of the PVC suspension production process (Case 4).

Meanwhile, as shown in Figure 6, the GWP and AP categories show significantly higher values. The total output registers a GWP of 496 PEI per day and an AP of 5140 PEI per day, the same values appearing in the total generated impact, which indicates direct emissions to the environment. These figures are mainly due to natural gas consumption to meet the process’s energy requirements, for which its combustion produces a high ${CO}_{2}$ and ${NO}_{x}$ footprint [43]. The acidification category (AP) is driven primarily by high nitrogen oxide (${NO}_{x})$ emissions, which, when dissolved in atmospheric water droplets, form nitric acid and acid rain [44].

When examining the output impacts per ton of product, these values decrease markedly. The GWP reaches 0.39 PEI per ton of product, while the output AP is 4.02 PEI per ton of product, with no change in the generated per-ton values. These per-ton values are calculated using the same production basis as the daily totals. This reflects that, thanks to the high production yield, the process maintains a low environmental footprint per unit of product.

### 3.3. Impacts According to the Energy Source Used by the Process

**Figure 7:**
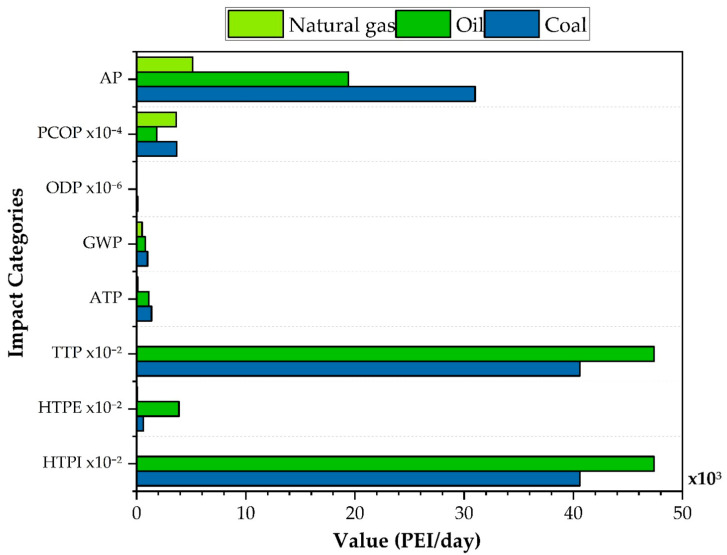
Output rate of PEI from energy usage comparison for PVC suspension production process.

Figure 7 shows the PEI for the toxicological and atmospheric categories for three energy sources used in the process (oil, coal, and natural gas) considering Case 4, which integrates the contributions of waste, product, and energy used for PVC production. In the human inhalation toxicity category (HTPI), natural gas presents the lowest toxicity value at 0.25 PEI per day, because its combustion is cleaner (primarily CO_2_ and water) and does not generate solid residues or large amounts of hazardous compounds. By contrast, oil reaches 474 PEI per day, since its combustion produces compounds such as heavy metals and chlorinated organic substances that are harmful to human health when they enter water or food or accumulate in the environment. Coal shows an intermediate value of 406 PEI per day, because although its residues are less soluble than those of oil, it contains sulfur compounds, traces of heavy metals, and organic by-products that can bioaccumulate and pose a risk if exposure is prolonged. The same ranking holds for terrestrial toxicity potential (TTP), as these categories relate to the release of the same substances.

In terms of human environmental exposure toxicity (HTPE), the values are 0.67 PEI per day for natural gas, 6.32 PEI per day for coal, and 38.8 PEI per day for oil. These results reflect the hazard posed by substances emitted by each fuel. Oil produces the highest impacts because its combustion generates complex compounds and fine particles that can be easily inhaled and cause harmful respiratory effects. Although coal also emits particles, it has a moderate value in this category, while natural gas again stands out as the best option due to its low release of harmful gaseous compounds.

Regarding aquatic toxicity potential (ATP), natural gas contributes 85.5 PEI per day, oil 1120 PEI per day, and coal 1370 PEI per day. These differences indicate each fuel’s capacity to release water-soluble substances that directly affect aquatic ecosystems, such as heavy metals, sulfides, and phenolic compounds.

Among all categories, the highest impact for all three fuels is acidification potential (AP), ranging from 5140 PEI per day for natural gas to 19,400 PEI per day for oil and 31,000 PEI per day for coal. This impact is related to the high emissions of pollutants such as sulfur oxide ${SO}_{x}$ and nitrogen oxide ${NO}_{x}$ generated during combustion, which react in the air to form strong acids such as sulfuric acid $H_{2}SO_{4}$ and nitric acid ${HNO}_{3}$. These acids are deposited as acid rain or dry deposition, affecting soils, plants, buildings, bodies of water, air quality, and ultimately human health through prolonged exposure [45].

In contrast, natural gas emits far lower amounts of sulfur oxides ${SO}_{x}$ and nitrogen oxides (${NO}_{x}$), whereas coal shows the worst performance, nearly doubling oil’s impact and sextupling gases. This is due to its high sulfur content, present in various forms such as pyrite and organic sulfur compounds, for which its combustion releases large quantities of sulfur dioxide ${SO}_{2}$. The concentration and type of sulfur in coal depend on its formation environment and subsequent processes, with the highest-sulfur coals originating in marine settings rich in sulfates and bacterial reduction, which produce minerals such as pyrite [46].

Regarding global warming potential (GWP), values rise from 496 PEI per day for natural gas to 788 PEI per day for oil and 1000 PEI per day for coal. This reflects that heavy and solid fuels generate greater amounts of carbon dioxide ${CO}_{2}$ when burned, contributing to the greenhouse effect [7]. In contrast, the photochemical oxidation potential (PCOP) and ozone depletion potential (ODP) categories show very low values. Both oil and gas register zero, while coal is only 0.01 PEI per day for ODP. For PCOP, the values are 0.36 PEI per day for gas, 0.18 PEI per day for oil, and 0.37 PEI per day for coal. These results indicate that these categories are not significantly affected by the compounds typically released during the combustion of these fuels.

## 4. Conclusions

The suspension PVC production process, integrated in terms of mass and energy with wastewater regeneration through reverse osmosis, biological treatment, and coagulation with aluminum sulfate, achieved a favorable environmental performance. In Case 1, considering only the waste streams, results of −3160 PEI/day (−2.47 PEI/t) were obtained, reflecting the high efficiency of the system in converting toxic VCM into PVC. Subsequently, when product flow and energy consumption were included (Case 4), the output PEI increased to 6230 PEI/day, reflecting the impact of high-energy-consumption stages such as drying, desorption, and regeneration [33,34,35]. The use of natural gas as the primary energy source proved key to limiting CO_2_ emissions (GWP of 496 PEI/day) and NO_x_ emissions (AP of 5140 PEI/day), owing to its lower CO_2_ and NO_x_ intensity relative to coal and fuel oil [43,44]. Additionally, the treatment of reject water using aerobic and anaerobic reactors allowed the retention and concentration of trace compounds such as PVA, initiator and VCM, minimizing human toxicity by ingestion (HTPI), human toxicity by inhalation (HTPE), and terrestrial toxicity (TTP) categories with negative generation rates.

However, two challenges remain that could be addressed in future work. First, residual outputs related to aquatic toxicity potential (ATP) per ton of product were identified; although this value is low (0.06 PEI/t), traces of PVA and organochlorine compounds require additional filtration and, if possible, the use of additives with lower halogen content to minimize this impact. Second, the ozone depletion potential (ODP) exhibited a value of 1.88 × 10^−3^ PEI/day, indicating the absence of a stage capable of neutralizing halogenated compounds. Therefore, it would be important to evaluate dehalogenation technologies or selective adsorbents for these contaminants. Moreover, future research should expand the analysis to a full life cycle assessment (LCA) covering use and end-of-life stages, and explore other energy sources, such as renewables, to further reduce the process GWP and AP. Overall, the system demonstrated that by combining process integration strategies with wastewater regeneration, it is possible to produce PVC in a considerably cleaner and more environmentally responsible manner. This approach represents a robust environmental alternative capable of handling hazardous substances, minimizing emissions, and optimizing water and energy use, aligning with current sustainable development goals and environmental regulatory requirements.

## Figures and Tables

**Table 1:** Overview of the analysis scope in different processes.

Process	Mass Integration	Energy Integration	Water Regeneration	Environmental Evaluation with WAR	Reference
PVC suspension				x	[25]
PVC pinch energy		x			[26]
PVC suspension—WEP			x		[27]
PET recycling					[28]
Continuous PP				x	[23]
PP injection molding	x		x		[29]
Integrated and regenerated PVC suspension	x	x	x	x	This work

**Table 2:** Contribution of each stage to the total PEI production rate (Case 1: waste leaving the process).

Stage	Output Rate of PEI (PEI/Day)	Contribution (%)
VCM polymerization	0.000	0.0
PVC purification	0.000	0.0
VCM recovery	0.000	0.0
PVC drying	0.050	5.5
Regeneration	0.858	94.5
Total	0.908	100

## Data Availability

The data will be made available upon reasonable request to the corresponding author (Á.D.G.-D.) due to privacy and legal reasons.

## Abbreviations

The following abbreviations are used in this manuscript:

PEI	Potential environmental impact;
PVC	Poly(vinyl chloride);
PE	Polyethylene;
PP	Polypropylene;
WAR	Waste reduction algorithm;
AP	Acidification potential;
GWP	Global warming potential;
PCOP	Photochemical oxidation potential;
ODP	Ozone depletion potential;
HTPI	Human toxicity potential by ingestion;
HTPE	Human toxicity potential by exposure;
TTP	Terrestrial toxicity potential;
ATP	Aquatic toxicity potential;
ORE	Output rate of PEI from energy usage (PEI/h);
TGR	Total generation rate of PEI (PEI/day);
TOR	Total output rate of PEI (PEI/day);
TPGS	Total PEI generated within a system per mass of products (PEI/t product);
TPLS	Total PEI leaving the system per mass of products (PEI/t product);
VCM	Vinyl chloride monomer;
COD	Chemical oxygen demand;
${BOD}_{5}$	Biochemical oxygen demand within five days.
